# Diode Laser Intended Frenotomy Procedure for High Frenal Attachment Causing Gingival Recession: A Case Presentation

**DOI:** 10.7759/cureus.70212

**Published:** 2024-09-25

**Authors:** Unnati Shirbhate, Komal Agrawal, Pavan Bajaj, Sourabh Shinde, Mrunal Meshram, Vaishnavi S Kayarkar, Punam S Patil

**Affiliations:** 1 Department of Periodontics, Sharad Pawar Dental College and Hospital, Datta Meghe Institute of Higher Education and Research, Wardha, IND; 2 Department of Oral Medicine and Radiology, Sharad Pawar Dental College and Hospital, Datta Meghe Institute of Higher Education and Research, Wardha, IND; 3 Department of Orthodontics and Dentofacial Orthopedics, Vidarbha Youth Welfare Society Dental College and Hospital, Amravati, IND; 4 Department of Pediatrics, Vidarbha Youth Welfare Society Dental College and Hospital, Amravati, IND

**Keywords:** diode, frenum, gingival recession, high frenum attachment, laser

## Abstract

The alveolar mucous membrane, the gingiva, and the underlying bone are fixed to the lip and cheek by the frenum, a fold in the mucous membrane. The surgical process known as a frenectomy involves completely removing the frenum that connects it to the bone. Traditional frenectomy involves using a blade, while a more recent technique is frenectomy assisted by a laser. When the frenum has an atypical connection, a labial or lingual frenectomy is performed. In this instance, there is an abnormal frequency of attachment, which puts the patient's appearance, causing midline diastema and plaque control, at serious risk and could result in mucogingival impairment. This case study describes how diode laser therapy was used to successfully treat a female patient who was suffering from sensitivity.

## Introduction

The frenum unites the mucous membrane of the alveolar process, gingiva, and underlying periosteum. A frenum or frena is a fold in the mucous membrane connecting the lip and cheek [1]. Frenum classification according to the insertion type includes the following: mucosal, gingival, papillary, and papilla penetrating. If the frenulum becomes overly tight for performing regular oral activities, then a simple surgical procedure known as the frenotomy will often be performed. It consists of resection and transposition of the frenal attachment. Dental treatment to obtain a perfect smile, which helps in a good look, has been considered due to esthetic reasons. Another disadvantage of attaching confront tissue tension may stem from a frenulum close to the gingival margin, leading to gingival recession. Dental lasers are currently being employed in many fields of periodontics, providing alternatives to conventional scalpel techniques. The use of diode laser frenotomy without infiltrated anesthesia is, currently, a novel technique. A shift of the marginal gingiva from its normal position on the crown of the tooth to a position coronal to the cementoenamel junction is defined as a recession of the gingiva or marginal gingiva [2]. Some of the clinical consequences that arise from this condition include cosmetic difficulties such as midline diastema, gingival recession and possible susceptibility to acquiring root caries, dentinal hypersensitivity, and even periodontal disease possibly [3]. By placing stress over the frenum and noting the movement of the papillary tip or the blanch indicative of ischemia, the preoperative appearance of the frenum can be appreciated. In such cases, one may consider the possibility of frenum revision. Therefore, one can perform a frenectomy using either laser, electrosurgery, or conventional scalpel surgery [1]. A disruption of the fine equilibrium of tissue mobilization and healing could predispose to gingival recession. Moreover, the probability of recession after surgery, which is a common complication, directly depends on the position as well as the degree of the frenal attachment. In particular, frenulum attachments that result in midline diastema, gingival recession, difficulty cleaning teeth in the region, compromise mentalis movements or are necessary for prosthetic purposes require frenectomy [4,5]. Initially, these procedures were carried out by conventional techniques using a scalpel. Laser electrocautery was considered a better technique in terms of hemostasis. Nowadays, frenotomy using a diode is preferred over a scalpel and electrocautery. Because of their improved visualization, precision, reduced hemorrhage, and fewer postoperative complications, lasers have a greater advantage over other techniques [6].

## Case presentation

**Figure 1:**
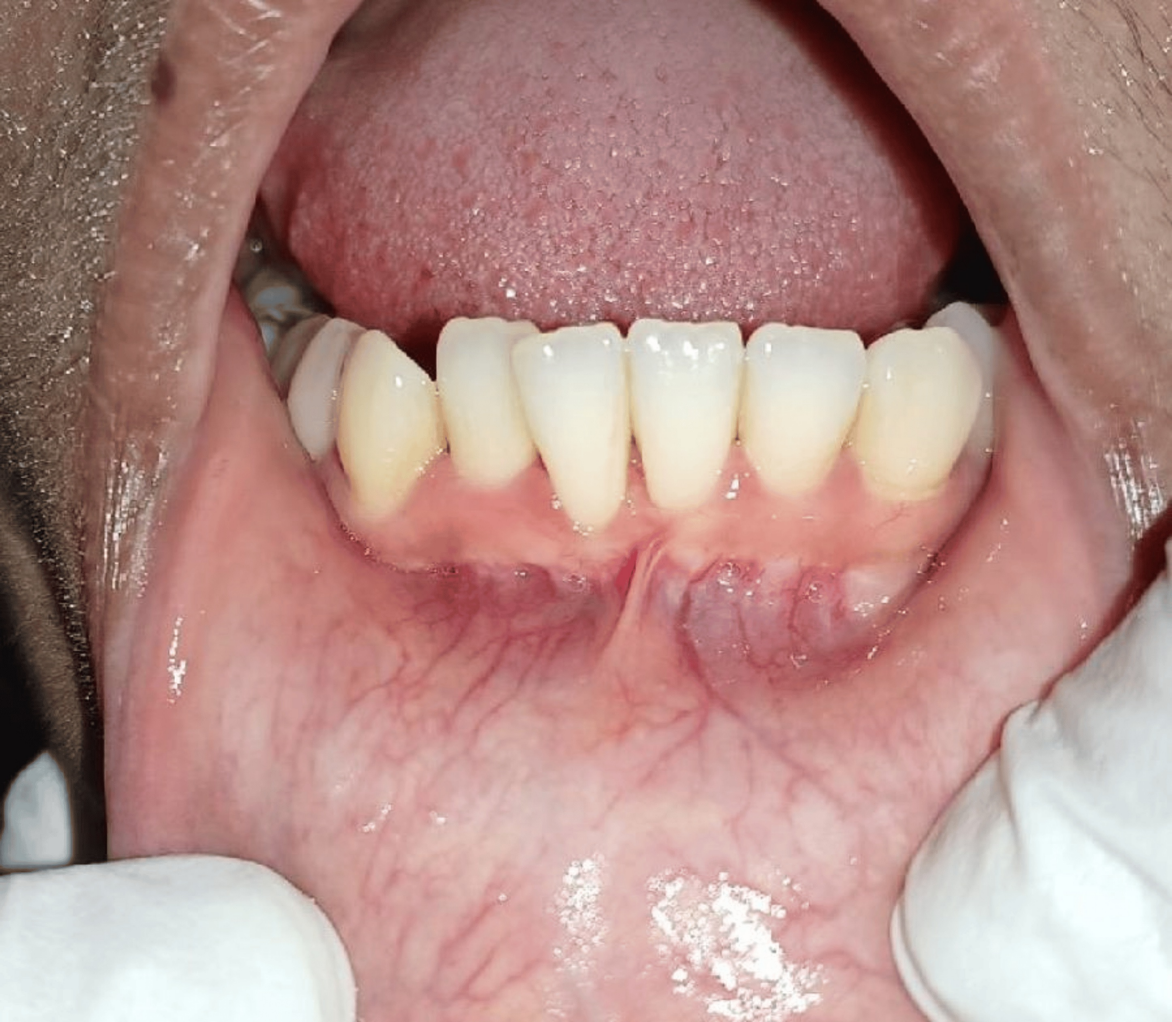
Initial preoperative view before scaling and polishing.

The 24-year-old female patient reported to the outpatient department of periodontics with a chief complaint of sensitivity and bleeding gums in the lower anterior region for the last two months. On clinical examination, crowding in mandibular anterior, lower labial high frenal attachment with class I gingival recession with mandibular right central incisor and thin gingival biotype with the same region shown in Figure 1.

**Figure 2:**
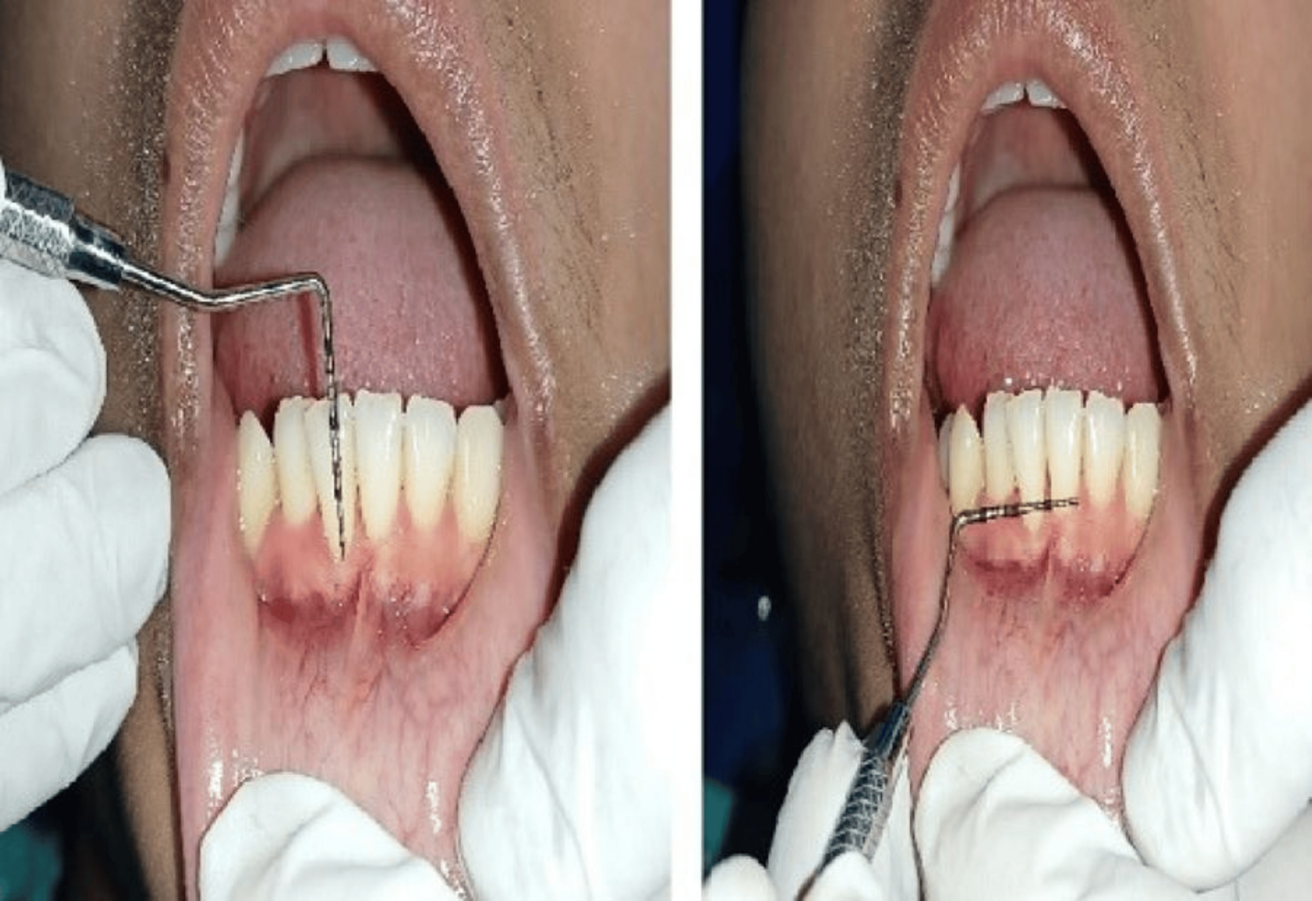
Preoperative view after seven days showing a high mandibular labial frenum attachment and gingival recession.

The patient was advised for scaling and polishing with desensitizing toothpaste for the same initially for seven days and surgical correction of high frenal attachment. After seven days of initial nonsurgical therapy (Figure 2), the patient came for the frenotomy procedure.

**Figure 3:**
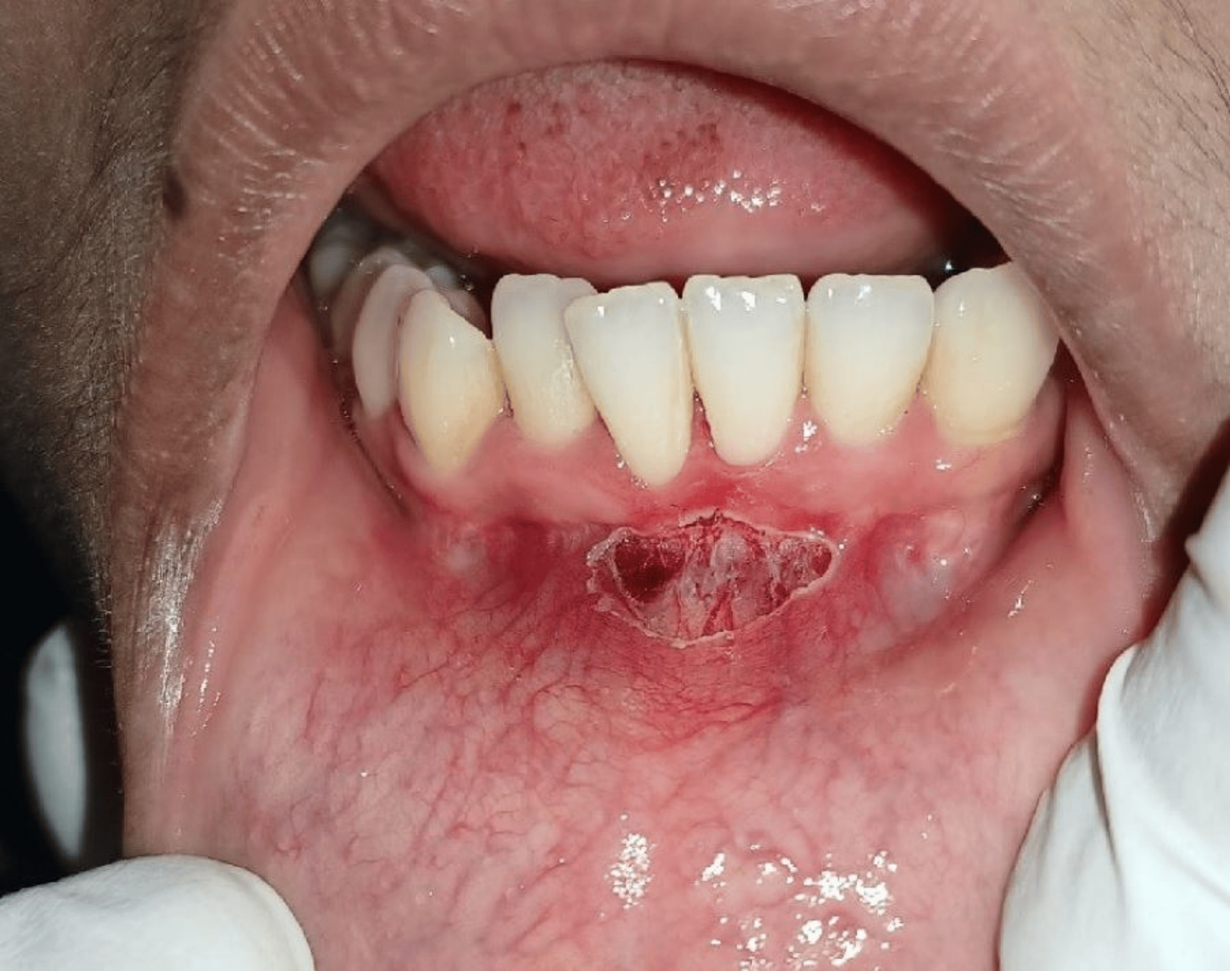
Frenotomy procedure was carried out using a diode laser.

The results of the hematological examination show that the hemoglobin level, bleeding time, clotting time, and random blood sugar level are all within normal ranges. The written informed consent was obtained. Under all aseptic conditions and precautions and local anesthesia, the diode laser frenotomy procedure was intended because of the patient's fear of seeing a scalpel and bleeding and to minimize pain and discomfort during the procedure. The frenotomy procedure has been carried out by using an 880 nm wavelength diode laser (Biolase, California, USA) with accompanying safety goggles and laser user protocols shown in Figure 3.

**Figure 4:**
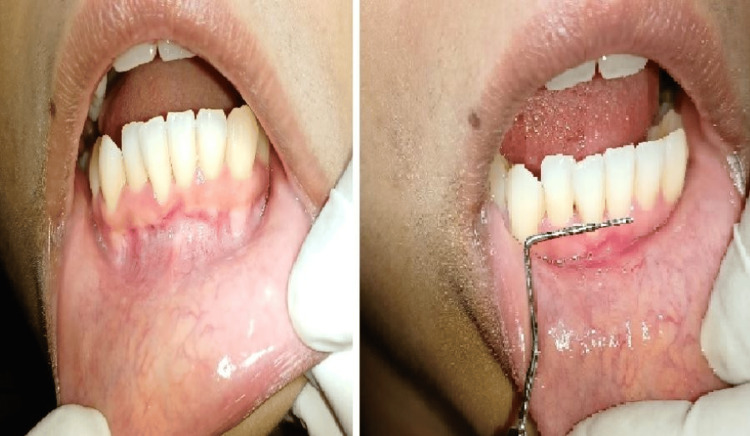
Postoperative view after three months showing complete satisfactory healing and no scar formation at the surgical site.

The patient was recalled for further periodontal evaluation after a week, where satisfactory healing was seen. After three months postoperatively, the patient underwent a review, revealing complete and satisfactory healing without any scar formation or discomfort, as shown in Figure 4.

## Discussion

Resection of the restrictive frenulum, which is known as the frenotomy, can bring about improvement of oral functions and general quality of life. This procedure requires the use of diode lasers, which have grown popular in this practice because of the accuracy they provide with very little intrusiveness. This discussion will explore the benefits of using a diode laser for frenotomy. In the current report, the patient underwent a frenotomy procedure using a diode laser. It was hoped this would correct symptoms associated with a tight frenum, feeding issues, and limited jaw mobility. Essentially several advantages are associated with the use of diode lasers in frenotomy. Diode lasers pose for a clean cut that is a little invasive to the other tissues in the area. This precision can decrease the amount of pain and suffering that patients may experience after the operation and enhance the general results [7]. Laser has the advantage of coagulating tissues during the process, thus minimizing bleeding and reducing the chances of infection. It is widely established that using lasers elicits less pain and discomfort as well as faster recovery time than conventional procedures [8]. Specifically, scalpel frenectomy is associated with after-surgery discomfort and pain, requires sutures, and can cause increased discomfort and problems for patients. For such procedures, laser surgery is a good substitute since, for most of them, sutures are not required or required minimally, surgeries are shorter, and after the operation, the patients experience less pain and discomfort, hence better acceptance [9].

Some other conventional approaches for frenotomy have been explained in the following techniques ahead: scalpel frenotomy is the traditional frenotomy that involves making an incision with a scalpel, which normally causes bleeding and typically requires stitches. Such a technique is associated with risks associated with a greater extent of tissue injury as well as postoperative pain. Another method is electrocautery. Electrocautery, on the other hand, is the utilization of electric currents in operations such as cutting and coating tissues. It can coagulate blood but may result in additional thermal injury to nearby tissues than the use of lasers [5]. Although possessing beneficial hemostatic and antimicrobial effects, the period of coagulation could be prolonged by the risk of heat-associated injury. Another method that is also applied is the cold knife technique. Cold knife techniques, while effective, result in excessive bleeding and need suturing. However, the traditional method may take a longer time to heal since there is more damage to the tissues and muscles as well as the chances of feeling more pain. Again, due to the application of diode lasers, there was little or no oozing, and there was prompt healing and less pain after the operation. The above outcome is consistent with earlier literature studies that show that diode lasers contribute to improvements in procedural efficiency and patient satisfaction [10]. Some possible risks that may be associated with using a diode laser for frenotomy include these problems; however, they can easily be prevented in instances where the correct technique is employed. However, some degree of skin care delegation, erythema, or impaired wound healing may occasionally be observed. In this case, there were no complications observed, and the patient was recovering fast.

## Conclusions

The following case demonstrates that the diode laser used during periodontal plastic surgery resulted in minimal discomfort and pain in anxious patients or patients with blood fear. The outcome after the frenotomy procedure was remarkable and minimized the chances of gingival recession, which could be extended in the future due to the persistence of mucogingival problems.
